# Targeted Next-Generation Resequencing of *F*5 Gene Identifies Novel Multiple Variants Pattern in Severe Hereditary Factor V Deficiency

**DOI:** 10.1155/2013/941684

**Published:** 2013-04-15

**Authors:** Piotr K. Janicki, Sonia Vaida, Hamid A. B. AL-Mondhiry

**Affiliations:** ^1^Laboratory of Perioperative Genomics, Department of Anesthesiology, Penn State University College of Medicine, MS Hershey Medical Center, H187, 500 University Dr, Hershey, PA 17033, USA; ^2^Division of Hematology-Oncology, Penn State University College of Medicine, MS Hershey Medical Center, Hershey, PA 17033, USA

## Abstract

The present study investigated the genetic defects underlying severe Factor V deficiency in a 26-year-old Columbian (South America) female and her immediate family (both parents and newborn child) by next generation sequencing (NGS) of the entire *F*5 gene locus. Five mutations in the coding sequence of *F*5, including three missense single-nucleotide variants (R2102H, R513K, D107H) and two synonymous variants (A135A , S184S), were identified and confirmed by the Sanger sequencing in the investigated proband (homozygote for all detected mutations), her parents, and her newborn child (all heterozygotic carriers for identified mutations). Each of the three missense variants was previously associated with separate phenotypes, including Factor V deficiency (R2102H), thrombosis (R513K) and frequent miscarriages (D107H). In addition, at least 75 additional single-nucleotide variants (including six novels) were identified in untranslated region of *F*5.

## 1. Introduction

Coagulation Factor V is a large 330-kD glycoprotein which consists of 2224 amino acid residues including a 28-residue leader peptide, which is structurally and functionally homologous to coagulation Factor VIII [1, 2]. The human Factor V gene (official name *F*5) maps to chromosome 1q23 and contains 25 exons (8). Factor V deficiency is a rare autosomal recessive disorder (incidence < 1 in 1 million), characterized by low levels of antigen and activity [3]. At present, more than one hundred deficiency-causing mutations in the *F*5 locus have been described, and although most of them are private, a few are common, being found in several individuals of both European, Middle-Eastern and Asiatic descents. In the present study, we used for the first time DNA next-generation sequence (NGS) analysis to detect mutation pattern in the entire *F*5 locus of a 26-year-old Hispanic parturient with severe Factor V deficiency, as well as in her asymptomatic parents and newborn baby. The relationship between combinations of mutations and clinical phenotypes was evaluated.

## 2. Case Presentation

**Figure 1:**
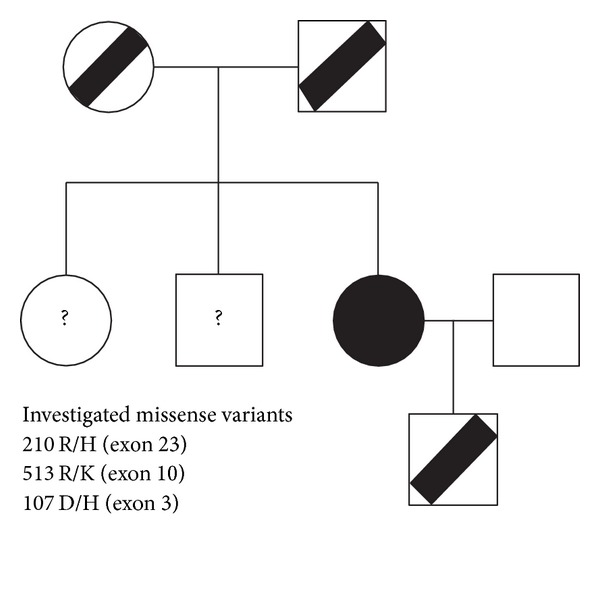
Segregation of *F*5 missense variants 2102 R/H (exon 23), 513 R/K (exon 10) and 107 D/H (exon 3). Half-filled symbols indicate heterozygotes for investigated mutatons. Completely filled symbols indicate homozygous for all investigated mutations (i.e., proband). Open symbols indicate no carrier of investigated mutations. Question marks indicate that genotyping has not been performed.

The study protocol was approved by IRB at PSU Hershey Medical College. It was performed in adherence to the tenets of the declaration of Helsinki. Written informed consent was obtained from all participants. The investigated patient was 26-year-old Hispanic (born in Columbia, South America) parturient (G4P1) with several bleeding episodes before and during present pregnancy, multiple fresh frozen plasma (FFP) transfusions, and a history of three miscarriages in the past. The patient was previously diagnosed clinically to have severe Factor V deficiency on the basis of several previous bleeding episodes and laboratory studies demonstrating coagulopathy with moderate to severe decrease in Factor V activity. The remaining past medical history was unremarkable. The family history revealed that both biological parents had no history of bleeding or other coagulation symptoms and had reported normal Factor V activity. In addition, she has two siblings, apparently without any clinical signs of coagulation disorders. In the course of the current pregnancy the patient delivered the healthy male newborn, who has not displayed, at the time of this analysis, any signs of coagulation abnormalities, besides decreased (36%) level of Factor V. For the purpose of this investigation, we collected the samples of blood (proband and newborn son) and saliva (both biological parents and proband's husband) for DNA analysis (Figure 1).

## 3. Mutation Analysis

Genomic DNA (gDNA) was extracted from venous EDTA-whole blood sample (proband) or cord blood (newborn child) employing membrane ultrafiltration method (FujiFilm Life Sciences distributed by Autogene, Holliston, MA, USA), according to the manufacturer recommendations. The saliva samples were collected from both parents and proband/s husband into Oragene container (DNA Genotek, Canada) and extracted according to the manufacturer recommendations. Subsequently two gDNA samples from the proband were submitted to Otogenetics Corporation (Norcross, GA, USA) for target capture and sequencing. Briefly, gDNA was subjected to agarose gel and optical density ratio tests to confirm the purity and concentration prior to Covaris (Covaris, Inc., Woburn, MA, USA) fragmentation. Fragmented gDNAs were tested for size distribution and concentration using an Agilent Bioanalyzer 2100 (Agilent Technologies, Santa Clara, CA, USA) and Nanodrop (Thermo Fisher Scientific, Wilmington DE, USA). Illumina libraries were made from qualified fragmented gDNA using NEBNext reagents (New England Biolabs, Ipswich, MA, USA) and the resulting libraries were subjected to exome enrichment using custom probes targeting 75 kb target on chromosome 1 (169, 481, 192–169, 555, 469 by GRCh 37, Hg19). The resultant libraries were tested for enrichment by qPCR and for size distribution and concentration by an Agilent Bioanalyzer 2100. The samples were then sequenced on an Illumina HiSeq2000 (Illumina, San Diego, CA, USA) which generated paired-end reads of 90 or 100 nucleotides. Data was analyzed for data quality, exome coverage, and exome-wide SNP/InDel using the platform provided by DNAnexus (DNAnexus, Inc, Mountain View, CA, USA). The detected polymorphisms in the coding sequence of the *F*5 locus were subsequently verified using classical Sanger sequencing using exon primers described by van Wijk et al. [4]. This analysis was performed by direct DNA sequencing using ABI Prism 3100 genetic analyzer (Applied Biosystems, Foster City, CA, USA).

**Figure 2:**
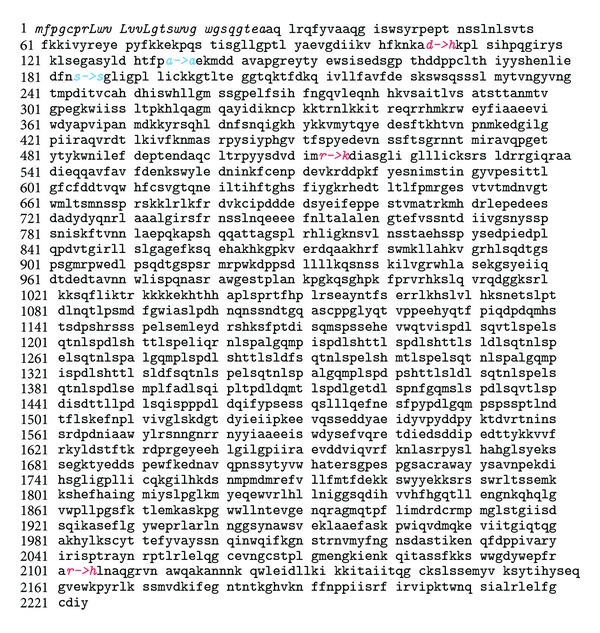
Amino acid sequence of human factor V deducted from the *F*5 DNA sequence of proband. Red fonts indicate missense mutations, and blue fonts indicate synonymous mutations. The sequence of first 28 amino acid fragments is given in italics, and the consecutive 25 exons are either underlined or not underlined (pair wise).

By NGS, we generated about 549 million bases of sequence as pair-end 90 or 100 nucleotide reads, 86% of which was able to align to human reference sequence. A total of 23% of these sequences mapped to the targeted region corresponding to 75 kb sequence of the *F*5 locus (NM_0001304.4), with 540-fold mean coverage (Table 1). At this depth of coverage, more than 95% of the target bases were covered to pass quality control filtering based on the PHRED score threshold of calling variants (PHRED > 30). Eighty high-confidence variants were annotated in the target region (3 nonsynonymous single-nucleotide variants and 2 synonymous single-nucleotide variants in the coding region of the target (Table 2), as well as 75 single-nucleotide variants in the noncoding sequence of the target (Table 3). The missense variants were located in the exon 3 (D107H), 10 (R513K), and 23 (R2102H) of the *F*5 locus (Figure 1), and the investigated patient was a homozygous carrier for all these variants. These variants were additionally confirmed by the Sanger sequencing and showed perfect match in two duplicate samples. The additional verification of the DNA samples from the proband's newborn son and our patient's biological parents performed using the Sanger sequencing revealed that all of them were heterozygous carriers for all 3 missense variants (Figure 2). No presence of these variants was shown in the husband of the patient (i.e., biological father of the newborn child).

## 4. Discussion

The current study presents several novel findings: (i) it employs NGS approach for molecular diagnosis of FV deficiency, including sequencing of both translated and untranslated regions of *F*5 locus; (ii) it diagnoses the inherited FV deficiency in the S. American Caucasian family of Hispanic ethnicity; and (iii) it depicts detailed genetic evidence for multiple, coinherited mutations in the *F*5 locus which may be responsible for opposite phenotype characteristics (i.e., increasing and decreasing thrombosis process). 

The results of the current study indicate that the investigated proband was a homozygotic carrier of three separate missense mutations in the coding sequence of *F*5. Surprisingly, however, only one of the 3 detected missense mutations (R2102H) was described previously (and called at that time, R2074H, based on different amino acid number order) by Schijver et al. (2002) in the context of the deficiency of the Factor V [5]. This substitution results in the replacement of an arginine (R) by a histidine (H) in amino acid position 2102 located in the C2-domain of Factor V and several lines of evidence reported previously support the notion that this sequence variant is causative for Factor V deficiency phenotype. Interestingly the remaining two detected missense mutations were reported to be associated mostly with either thrombosis [6–8] or increased risk of preterm delivery (D107H) [9]. It is noteworthy that two missense point mutations which were detected in the current proband were previously described in different populations (R2102H in Tunisian population and R513K in Thai, Chinese and Sub-Saharan populations). Although Factor V deficiency and its causative mutations were reported previously from European Caucasians, it is, to our knowledge, the first detailed description of causative mutation in *F*5 locus in a family from South America and/or Hispanic ethnicity.

In addition, we established that the investigated proband was a homozygous carrier for two synonymous single-nucleotide variants: A135A (rs6029) and S184S (rs6022) previously reported in the online SNP Medline database. There is no information about the potential phenotypic significance of these mutations. In addition to the previously described single-point mutations in the coding region of *F*5 gene, the analysis of the NGS data from the proband established a presence of additional 75 polymorphisms in the untranslated sequence of the *F*5 locus (3 in 5′-UTR part, 71 in the intronic part, and 1 in the 3′-UTR part of the gene) (Table 3). Six of these variants represent newly discovered variants, not presented previously in the SNP Medline database. The types of detected variants include single-nucleotide variants (4 insertions and 4 deletions), as well as 3 additional short indels. The phenotypic significance of these polymorphisms for the Factor V function remains unknown.

The most recently (Oct 2012) accessed Human Genome Mutation database (www.hgmd.org) lists 145 missense mutations in the *F*5 locus associated with altered function of Factor V. From this list, 94 mutations represent single-point mutations, from which approximately 80 have been strongly associated with Factor V deficiency. In this respect, the present study does not add new mutations to this list but confirms the fact of previously described coinheritance of several separate mutations in the *F*5 locus [10, 11], and more importantly provides an example of co-inheritance of *F*5 mutations with presumably opposite phenotypic coagulation characteristics. Similar situation (i.e., coinheritance of polymorphic variants from which one is associated with decreased Factor V activity and other with increased thrombosis) was described previously for much more frequent prothrombotic Leiden mutations, or more recently, promoter −426 G/A polymorphism [12, 13]. Our finding confirms that other prothrombotic mutations in the *F*5 gene locus may be independently inherited in one patient.

The present study exemplifies the use of NGS approach for detailed diagnosis of the specific clinical pathology and identification of causative mutations for rare genetic disorder. This approach has been recently advocated for both diagnosis and therapy [14]. The obtained genetic data for this cases were successfully used clinically for the peripartum anesthetic management of the previously described patient [15]. Comprehensive genetic analysis through NGS based approaches will increasingly be helpful in establishing the diagnosis of Factor V deficiency (or other genetic coagulation disorders) and thereby improve patient management.

## Figures and Tables

**Table 1:** Summary of coverage for analyzed samples by next generation sequencing method.

Sample	Average target coverage	Total bases	Total reads	Mapped reads	Bottleneck score
Original proband DNA sample	547	583,280,200	5,832,802	4,964,887	31.81
Duplicate	534	549,426,400	5,494,264	4,720,781	32.81

**Table 2:** Mutations observed in coding sequences (CDS) of *F*5 gene in the investigated proband and her blood relatives.

Patient	Bleeding phenotype	FV activity (%)	Variants in CDS of *F*5 gene	Mature FV protein variants	SNPs (ID)
Female proband	Epistaxis multiple miscarriages	0–4%	Homozygote for nonsynonymous variants:		
			169486641 G > C	2102 R/H (exon 23)	No dbSNP ID
			169519112 C > T	513 R/K (exon 10)	rs6020
			169541513 C > G	107 D/H (exon 3)	rs6019
			Homozygote for synonymous variants:		
			169529973 C > T	135 A/A (exon 4)	rs6029
			169529826 C > A	184 S/S (exon 4)	rs6022

Newborn son of proband	No bleeding	36%	Heterozygote for nonsynonymous variants:		
			169486641 G > C	2102 R/H (exon 23)	No dbSNP ID
			169519112 C > T	513 R/K (exon 10)	rs6020
			169541513 C > G	107 D/H (exon 3)	rs6019
			Heterozygote for synonymous variants:		
			169529973 C > T	135 A/A (exon 4)	rs6029
			169529826 C > A	184 S/S (exon 4)	rs6022

Mother of proband	No bleeding	Reported normal	Heterozygote for nonsynonymous variants:		
			169486641 G > C	2102 R/H (exon 23)	No dbSNP ID
			169519112 C > T	513 R/K (exon 10)	rs6020
			169541513 C > G	107 D/H (exon 3)	rs6019
			Heterozygote for synonymous variants:		
			169529973 C > T	135 A/A (exon 4)	rs6029
			169529826 C > A	184 S/S (exon 4)	rs6022

Father of proband	No bleeding	Reported normal	Heterozygote for nonsynonymous variants:		
			169486641 G > C	2102 R/H (exon 23)	No dbSNP ID
			169519112 C > T	513 R/K (exon 10)	rs6020
			169541513 C > G	107 D/H (exon 3)	rs6019
			Heterozygote for synonymous variants:		
			169529973 C > T	135 A/A (exon 4)	rs6029
			169529826 C > A	184 S/S (exon 4)	rs6022

Proband's husband	No bleeding	Reported normal	No above CDS variants detected in *F*5	—	—

**Table 3:** Summary of observed variants in noncoding sequences of *F*5 gene in investigated proband.

Type of variant	Type of carrier	Position-R	Position-L	Reference base	Variant base	SNPs (ID)
Variants in 5′-UTR						

SNP	Hom	169556050	169556051	c	t	rs2269648
SNP	Hom	169556152	169556153	g	t	New variant
INS	Het	169556812	169556812		a	rs58897818

Intronic variants						

SNP	Hom	169486641	169486642	g	c	rs9332666
SNP	Hom	169490392	169490393	g	c	rs2420370
SNP	Hom	169491555	169491556	g	a	rs2420371
SNP	Hom	169496536	169496537	g	c	New variant
SNP	Hom	169498056	169498057	a	g	rs2420372
SNP	Hom	169516507	169516508	g	a	rs12046953
SNP	Hom	169517159	169517160	c	t	rs12026997
SNP	Hom	169517385	169517386	a	g	rs12044669
SNP	Hom	169517904	169517905	g	a	rs2420374
SNP	Hom	169518703	169518704	t	c	rs58875232
DEL	Het	169518885	169518886	a		rs55717622
SNP	Hom	169519417	169519418	g	a	rs7537742
SNP	Hom	169519765	169519766	a	g	rs13306331
SNP	Hom	169520098	169520099	a	g	rs10800456
SNP	Hom	169521553	169521554	g	a	rs2213868
SNP	Hom	169521733	169521734	c	t	rs9332582
SNP	Hom	169523346	169523347	a	g	rs7555832
SNP	Hom	169523389	169523390	t	c	rs11577059
SNP	Het	169523395	169523396	a	t	New variant
SNP	Het	169523396	169523397	a	g	New variant
DEL	Hom	169523397	169523398	t		rs116132528
INS	Het	169524860	169524860		cac	New variant
SNP	Het	169524865	169524866	a	g	New variant
SNP	Hom	169525312	169525313	c	t	rs9332579
INS	Het	169525558	169525558		ctctggc	rs16684
SNP	Het	169525680	169525681	c	a	rs115199761
SNP	Hom	169525766	169525767	t	c	rs2239853
INS	Hom	169526266	169526266		a	rs9332577
SNP	Hom	169526300	169526301	c	t	rs4656688
SNP	Hom	169526367	169526368	a	g	rs4656689
SNP	Hom	169526425	169526426	g	t	rs4656188
SNP	Hom	169526601	169526602	g	c	rs1894697
SNP	Hom	169526646	169526647	a	g	rs1894698
SNP	Hom	169526950	169526951	c	g	rs1894699
SNP	Hom	169527226	169527227	a	g	rs1981491
DEL	Hom	169527470	169527471	a		rs3835454
SNP	Hom	169528075	169528076	c	g	rs9332570
INS	Het	169528255	169528255		aaa	rs58738850
SNP	Hom	169528580	169528581	c	t	rs6012
SNP	Hom	169528722	169528723	c	t	rs6427201
SNP	Hom	169528830	169528831	c	t	rs6427202
SNP	Hom	169529031	169529032	a	c	rs6427203
SNP	Hom	169529132	169529133	c	t	rs6699691
SNP	Hom	169529138	169529139	c	t	rs58931047
SNP	Hom	169530070	169530071	a	c	rs7545236
SNP	Hom	169530077	169530078	g	t	rs7523043
SNP	Hom	169530093	169530094	t	c	rs7534848
SNP	Hom	169530176	169530177	c	g	rs7522982
INS	Hom	169530532	169530532		a	rs5778621; rs77192101
SNP	Hom	169530586	169530587	t	c	rs1894701
SNP	Hom	169531442	169531443	t	c	rs7540556
SNP	Hom	169531571	169531572	t	c	rs4656690
SNP	Hom	169533266	169533267	g	a	rs6678795
SNP	Hom	169533744	169533745	t	a	rs724509
SNP	Hom	169534028	169534029	t	c	rs724507
SNP	Hom	169534966	169534967	t	a	rs2040443
SNP	Hom	169535353	169535354	c	g	rs6685578
SNP	Hom	169536167	169536168	t	c	rs2213869
SNP	Hom	169536650	169536651	g	a	rs2187955
DEL	Het	169536796	169536798	tt	t	New variant
SNP	Hom	169537678	169537679	a	g	rs6670678
SNP	Hom	169538466	169538467	g	a	rs9287095
SNP	Hom	169538544	169538545	c	t	rs2298908
SNP	Hom	169538603	169538604	t	g	rs2298906
SNP	Hom	169539348	169539349	t	g	rs6663533
SNP	Hom	169543263	169543264	c	a	rs10800457
SNP	Hom	169545413	169545414	a	g	rs6677374
SNP	Hom	169549775	169549776	t	c	rs10753787
INS	Het	169551560	169551560		a	rs56901113
SNP	Hom	169554058	169554059	c	g	rs3753305

Variants in 3′-UTR						

SNP	Hom	169479974	169479975	c	t	rs970740

Hom: homozygote, Het: heterozygote, SNP: single-nucleotide polymorphism, Del: deletion, and Ins: insertion
